# Factors hindering midwives’ utilisation of alternative birth positions during labour in a selected public hospital

**DOI:** 10.4102/phcfm.v11i1.2071

**Published:** 2019-09-17

**Authors:** Maurine R. Musie, Mmapheko D. Peu, Varshika Bhana-Pema

**Affiliations:** 1 Department of Nursing Science, University of Pretoria, Pretoria, South Africa

**Keywords:** Alternative birth positions, Factors, Midwives, Labour, Utilisation

## Abstract

**Background:**

An evidence-based practice suggests that the birth position adopted by women during labour has a significant impact on the maternal and neonatal birth outcomes. The birth positions are endorsed by guidelines of maternity care in South Africa, which documented that women in labour should be allowed to select the birth position of their choice, preferably alternative birth positions (including upright, kneeling, squatting and lateral positions) during labour. Thus, the lithotomy birth position should be avoided. However, despite available literature, midwives routinely position women in the lithotomy position during normal vertex births, which causes several adverse maternal outcomes (namely prolonged labour, postpartum haemorrhage) and adverse neonatal outcomes (such as foetal asphyxia and respiratory compromise).

**Aim:**

The aim was to explore and describe factors hindering midwives’ utilisation of alternative birth positions during labour in a selected public hospital.

**Setting:**

A public hospital in the Tshwane district, Pretoria were used in the study.

**Methods:**

This study used the qualitative, exploratory and descriptive research design. This design gathered quality information on factors hindering midwives’ utilisation of alternative birth positions during labour in a selected public hospital.

**Results:**

The study revealed the following themes: (1) midwives’ perceptions on alternative use of birth positions and (2) barriers to utilisation of alternative birth positions. The themes were discussed and validated through the use of a literature review.

**Conclusion:**

The lack of skills and training during the midwifery undergraduate and postgraduate programme contributes to the midwives being incompetent to utilise alternative birth positions during clinical practice.

## Introduction

Before colonisation in Africa, it is evident that women were giving birth in various alternative birth positions, such as sitting, upright position, squatting, kneeling, using hands and knees and the left lateral birth positions. These positions were common birth practices that usually occurred in a home setting.^1^ The current literature supports an evidence-based birth position that is beneficial to women.^2^ The World Health Organization^3^ endorses the use of alternative birth positions which are associated with favourable maternal and childbirth outcomes. Within the South African context, the guidelines for maternity care in South Africa supported by the National Department of Health endorse the use of alternative birth positions during labour.^4^ The guidelines stipulated that women should be positioned in a birth position of their choice, preferably in alternative birth positions; the lithotomy birth position should be avoided in the first and second stages of labour.^4^ However within the South African context, midwives are still utilising the lithotomy birth position irrespective of the available evidence-based literature.

The use of the lithotomy birth position during labour is associated with negative maternal and neonatal outcomes. During labour in the lithotomy birth position, a woman lies flat on her back, with hips and knees flexed, thighs apart and legs supported in raised stirrups. The gravid uterus compresses the abdominal aorta vessels, thus obstructing blood flow to the uterus, resulting in the detrimental effects of maternal hypotension and reduced foetal oxygenation.^5,6^ Secondly, as the woman is flat on her back, trying to bear down against the force of gravity, foetal head descent is inhibited, therefore further exposing the woman to other envisaged obstetric complications such as the prolonged duration of labour that increases the chance of her being booked for emergency caesarean section or instrumental delivery to take place (vacuum delivery).^5,6^ Lastly, the woman might sustain perineal injuries involving anal sphincter, perineal tears, episiotomy and the increased rate of analgesia request associated with birth on lithotomy.^2,5,6^ Neonates born with the lithotomy position are at risk of reduced Apgar scores, birth asphyxia and increased intensive care admissions.

In contrast, evidence supports the use of alternative birth positions during the first and second stages of labour.^2,7^ These positions facilitate labour through normal physiological functioning that utilises the force of nature and gravity and are associated with optimal maternal and neonatal outcomes.^2,7^ A systematic review conducted found x-ray results showing the actual increase in pelvic diameters (antero-posterior and transverse pelvic outlet diameters) when a woman adopts alternative birth positions such as upright, squatting and kneeling during childbirth compared to the lithotomy position. Increase in pelvic diameter assists with the facilitation of labour and childbirth.^8^ During labour on alternative birth position, the women experiences an increase in the frequency and intensity of uterine contractions, resulting in foetal descent and cervical dilation resulting in birth of the foetus.^8,9^ In addition, the utilisation of these positions enhances progression and prevents prolonged duration of labour, thus reducing complications such as postpartum haemorrhage.^1^ Furthermore, alternative birth positions are associated with lower incidence rates of performing episiotomy, less perineal tears and less use of instrumental deliveries.^5,10^

Midwives’ lack of skills in assisting women during labour has been associated with the cause of ‘avoidable causes’ to maternal mortality in South Africa. The mortality rates are supported by recent statistics of the National Committee on the Confidential Enquiries into Maternal Deaths.^11^ The statistics indicated that over 39% of all maternal deaths are from avoidable causes, consequently as a result of midwives’ lack of knowledge and skills in assisting women during labour and childbirth. Maternal deaths during pregnancy, childbirth and the puerperium seem to be a significant public health issue in South Africa. A global strategy was implemented, in essence to reach the primary target of 2030 sustainable goal agenda; and the global reduction of maternal mortality ratio to less than 70 per 100 000 live births in South Africa. The strategy indicates that it is mandatory for midwives to be skilful in managing labour and contribute to the reduction of maternal mortality rate.^12^ The National Committee on the Confidential Enquiries into Maternal Deaths^11^ indicates that reduction in maternal mortality will be achieved by increasing the proportion of skilled birth attendants, for instance, midwives in labour units. The proposed solution is alternative birth positions, which can be used to address the avoidable causes to maternal mortality. These positions are evidence-based and are associated with optimal maternal and neonatal outcomes.

Studies by Nieuwenhuijze reveal that women’s birth experience is associated with short- and long-term implications on women’s health and well-being.^13,14,15^ Apart from that, Niewenhuijze^14^ demonstrates that women who are given a sense of control on the birth position of their choice develop self-esteem and a feeling of competence. Therefore, it means women need to be given a chance of having their sense of control and being involved in decision-making, especially on the type of birth position they would want to adopt during labour. There are also negative birth experiences that are likely to affect women’s emotional well-being. Some of the women are likely to present with depression, which is likely to affect the bond between the mother and the baby. Consequently, this leads to the avoidance of subsequent pregnancies and electing caesarean section/s without any apparent reason in the future.^14^

Despite this, midwives should always play a significant role, which might be used to prevent the identified negative experiences associated with birth outcomes. Based on this, one of the roles of the midwives is to provide women-centred care that enables women to adopt birth positions they are comfortable with, and that are likely to contribute to their self-esteem and their well-being.^13^ However, regardless of the benefits of alternative birth positions, women are not aware of a variety of alternative birth positions available to them.^1,15^ Therefore, this paper is about the factors hindering midwives’ utilisation of alternative positions during labour.

### Problem statement

The current practice within the South African context condones the use of the lithotomy birth position during labour. Midwives are routinely positioning pregnant women in the lithotomy position during the first and second stages of labour as noted by the researcher. Women are restricted in lithotomy or supine positions during foetal monitoring for a prolonged duration. During the second stage of labour, women bear down flat on their backs exposing themselves to several negative outcomes including perineal injury and postpartum haemorrhage as evident in the maternity case records. Most significantly midwives lack skill in the utilisation of alternative birth positions. Literature highlights the associated several negative maternal birth outcomes to the lithotomy birth position, including prolonged labour, perineal injuries and postpartum haemorrhage.^5,6,16^ A study found an association between women who are positioned in the supine/lithotomy position during pregnancy and increased stillbirth rates.^16^

That said, guidelines for maternity care in South Africa endorse alternative birth positions that women in labour are allowed to select. Preferably women should be allowed to birth in alternative birth positions (including upright, kneeling, squatting and lateral positions) during the first and second stages of labour.^4^ Allowing a woman to adopt alternative birth positions during the first and second stages of labour facilitates the descent of the foetus through gravity and is associated with optimal maternal and neonatal outcomes.^5^ These positions are also known to reduce the risk of maternal postpartum complications such as postpartum haemorrhage and perineal injury.^7,17^

Irrespective of available evidence-based guidelines on alternative birth positions, midwives continue utilising the lithotomy position during labour. Furthermore, the midwives do not seem to be aware of the effects associated with the lithotomy position as they continue and vouch for the practice. Therefore, the researcher intends to explore and describe the reasons why midwives continue to position women in the lithotomy birth position and disregard alternative birth positions during the management of the first and second stages of labour.

### Aim of the study

To explore and describe factors hindering midwives’ utilisation of alternative birth positions during labour at a selected public hospital.

## Research method and design

The study used qualitative, exploratory, descriptive and contextual design. This design was used purposively to explore and describe factors hindering midwives’ utilisation of alternative birth positions during labour at a selected public hospital.

### Setting

The study was conducted at a specific public hospital located in the Central Tshwane sub-district with an estimated population of more than 400 000 people. This hospital is a level-one district hospital that provides 24-hours low risk and emergency services to urban and rural areas surrounding the hospital. The hospital also serves as a referral hospital for other level hospitals and the clinics nearby. The bed occupancy in the labour ward is 20. Staffing in the labour ward consists of three categories of nurses registered by the South African Nursing Council. These are advanced midwives, professional nurses and staff nurses. The average number of nurses in each shift for both day and night ranges between four midwives and two staff nurses. The target population was the midwives who conduct normal vaginal births in the hospital. The ward birth statistics were approximately more than 300 birthing women per month.

### Study population and sampling

The study population included professional nurses with midwifery training who completed either the four-year degree or 3-year diploma course and advanced midwives with a speciality in midwifery registered by the South African Nursing Council. This equated to 30 midwives working in the labour ward.

Purposive sampling was used to select midwives who met the inclusion criteria. The criteria included qualified midwives currently working in the labour ward and responsible for conducting normal vertex deliveries with a minimum experience of 1 year working in the labour ward. The midwives who participated in the study were recruited based on availability and eligibility to participate. Data saturation occurred after conducting 20 interviews with the midwives who were willing to partake in the study based on the inclusion criteria.

### Data collection

Data were collected in a private room at the selected hospital in Tshwane. Face-to-face semi-structured interviews were conducted. The interview had one central question and probing follow-up questions. The central question asked was:

What are the factors hindering midwives’ utilisation of alternative birth positions during labour in a selected public hospital?

Interviews were digitally recorded, and the researcher took field notes. Permission to use a tape recorder was obtained from the participants to enable the extraction researcher and the research assistant to transcribe the information word for word to ensure accuracy. Each interview lasted between 30 and 50 minutes. The researcher probed participants until no new information emerged and stopped interviews when participants indicated that there were no further inputs. At the end, the participants were thanked for their participation.

### Data analysis

The data were analysed using Tesch’s method of data analysis. The eight steps of Tesch’s method enabled the researcher to analyse the data using open coding systematically to analyse and code the data. This method of data analysis was chosen due to its ability to convert raw written and audiotaped data into a more narrative form.^18^ Data were transcribed verbatim and then coded by the researcher, and assisted by the supervisor. The research supervisor was provided with printed transcripts and voice recordings. Then consensus was reached between the researcher and research supervisor regarding the emerging two themes.

### Trustworthiness

Trustworthiness is a way of ensuring data quality and is enhanced as explained according to a model by Lincoln and Guba (1985).^19^ The following criteria of trustworthiness were applied in this study: credibility, conformability, transferability and dependability.

Credibility was ensured through prolonged engagement between the researcher and participants to build trust and rapport, allocating adequate time to collect data and stay in the field until data saturation was reached.^19^ A dependability audit was conducted with the assistance of an experienced researcher (supervisor) in qualitative research, who followed the entire progression of the study to confirm the findings. After data were collected, the supervisor was given transcripts to read through and provide input on interpretations already formulated by the researcher.^19^ Confirmability was presented through strategies of member checking.^19^ The researcher provided feedback to the participants about emerging interpretations, for the participants to confirm the accuracy of the collected data. Lastly, transferability was maintained through thick description, and the researcher searched extensive literature to support the phenomena being studied, as already highlighted in the background section of the study.^19^

### Ethical considerations

Ethical clearance was obtained from the Research Ethics Committee at the University of Pretoria (ethics number-133/2018). Permission to conduct the study was sought from the Chief Executive Officers of the respective hospital. Informed consent was obtained from the participants. Ethical principles that were considered included beneficence, justice and respect for human dignity. These were maintained throughout the study.

## Results

Twenty midwives participated in the study. The majority of the participants were females aged 23–60 years. Participants with a postgraduate qualification in advanced midwifery were 7, compared to 13 professional nurses with midwifery training who were interviewed. The participants’ work experience varied from 1 year to 18 years in labour wards. Table 1 reflects factors affecting midwives’ utilisation of alternative birth positions during labour at a selected public hospital. Data from the interviews revealed two main themes: midwives’ perceptions of alternative birth positions and barriers to the utilisation of alternative birth positions.

**TABLE 1:** Themes, sub-themes on the factors hindering midwives’ utilisation of alternative positions during labour in a selected public hospital.

Themes	Sub-themes
1. Midwives’ perceptions of alternative birth positions	1.1 Midwives’ personal convenience and comfortability 1.2 Women’s choice of birth position
2. Barriers to utilisation of alternative birthing positions	2.1 Lack of necessary skills and training 2.2 Lack of facilities and equipment 2.3 Communication difficulties between midwife and women

### Theme 1: Midwives’ perceptions of alternative birth positions

Midwives’ perception of alternative birth positions is the first identified theme. The perception of midwives refers to the preconceived psychological perception and thoughts that midwives possess in relation to alternative birth positions. Two sub-themes were explained under this theme: midwives’ personal convenience and comfortability and women’s choice of birth position.

#### Midwives’ personal convenience and comfortability

The midwives in this study preferred the lithotomy position as compared to alternative birth positions. Alternative birth positions, also known as non-supine positions of side lying, kneeling and squatting, were preferred to a lesser extent by the midwives. The reason given for their preference of the supine/lithotomy when assisting a delivery was that the position provides a good view of the perineum, ease of labour monitoring and minimising the midwives’ physical strain during the birth. These views depicted the lithotomy position as appropriate and comfortable for the midwives. Most of the midwives were aware of the disadvantages of the lithotomy birth position but still prefer utilising the position because they find it comfortable and familiar to themselves:

‘I would like to think what hinders the midwives from using alternative birth positions is convenience; it is more convenient for the midwife to have the woman on lithotomy position, although it is not the best position to use. If you become too controlling as the midwife to patient, then a lot of women get perineal tears. We need to position women on positions that come to them naturally …’ (Participant 7, female, 55 year’s, Advanced midwife & 8 year’s midwifery experience)

‘We utilise the lithotomy position because it favours the midwife most of the time, it is easier for me and has no benefit to the birthing woman. My view on alternative birth positions it can be done only if the midwife is comfortable with it …’ (Participant 8, female, 46 year’s, Advanced midwife & 14 year’s midwifery experience)

Another midwife states procedural reasons for using lithotomy:

‘I place woman on lithotomy because when I need to perform episiotomy it is much [*more*] comfortable for us; if she is in squatting how are you going to perform episiotomy. We as midwives we are in control of the labour when we do what is comfortable for us …’ (Participant 15, female, 46 year’s, Advanced Midwife & 12 year’s midwifery experience)

#### Women’s choice of birth position

The midwives in the study differed in opinion of whether women should be given a choice of birth position or not. Some of the midwives agreed to involve a woman in the decision of their child’s birth. While most of the midwives verbalised that they did not have time to teach mothers about alternative birth positions.

Participants’ mentioned reasons for utilising the lithotomy position as workplace culture:

‘We always found the lithotomy being used here in this institution. To answer the question no we never give the woman a choice of birth position, the women are not literate enough to know their rights or maybe the information that needs to be given to them’ To be honest we don’t even inform them at all of the birth positions available, they are not given an option because we are not going to go with her option we only use birth positions that suit us not the patient …’ (Participant 1, female, 36 year’s; Advanced Midwife & 9 year’s midwifery experience)

‘I place the woman on lithotomy position because it is what I found being done in the unit. I think it is a culture of this unit and I know I was taught on other birth positions during studies, but I have never practised it. (I guess we are just joined what the Romans do in Rome, so I adopted the culture) …’ (Participant 9, female, 24 year’s, Professional nurse & 3 year’s midwifery experience)

Another midwife differed in perception:

‘I think they should be given a choice of birth position, as it will make them comfortable instead of forcing them to use one birth position.’ (Participant 2, female, 30 year’s, Professional nurse & 4 year’s midwifery experience)

Notably, an exciting finding was that there was no substantial difference between preferences of utilising alternative birth positions during labour between the general midwives and advanced midwives. The advanced midwives’ scope of practice requires them to implement evidence-based clinical practices. However, even advanced midwives are not competent in the utilisation of alternative birth positions after acquiring a postgraduate advanced qualification.

### Theme 2: Barriers to utilisation of alternative birth positions

The second identified theme is barriers to the utilisation of alternative birth positions. The following three sub-themes emerged under the identified theme: lack of necessary skills and training, lack of facilities and equipment, and communication difficulties between midwives and women. Barriers to the utilisation of alternative birth positions in this study indicate the numerous barriers identified during clinical practice, which hinder midwives’ utilisation of alternative birth positions.

#### Lack of necessary skills and training

The lack of necessary skills and training was identified as the first sub-theme. Most midwives were concerned that they do not possess the necessary skills and training to conduct alternative birth positions and are not confident enough with the skill. The midwives argued that the alternative birth position was taught in theory during undergraduate training. However, they were unable to grasp the skill and competence on how to practically position the women in alternative birth positions.

Poor skills development and training were discussed as follows:

‘Yes, we were taught about alternative birth positions, but we do not have the skill to use alternative birth positions. When I attended my undergraduate training at learning institutions, lecturers did not teach alternative birth position[*s*] in depth. As students, we were allocated in a different hospital and what I observed in the labour ward alternative birth positions are not practised. Therefore all we have been exposed to from undergraduate study is [*the*] lithotomy position. As midwives, we have built skill in mastering the lithotomy position, and we do not have confidence to offer alternative birth positions to women because we lack skill.’ (Participant 1, female, 36 year’s; Advanced Midwife & 9 year’s midwifery experience)

Another participant mentioned the lack of practice as a barrier to utilisation:

‘The midwives do not practice what they learnt in theory if we don’t practice what we learnt in undergraduate class the skill fades away. We all know that […] if we learn and practice, we become competent and become comfortable to use other birthing positions. I think many midwives do not practice alternative birth positions because they are not confident in utilising those positions …’ (Participant 20, female, 40 year’s, Advanced Midwife & 15 year’s midwifery experience)

#### Lack of facilities and equipment

The midwives differed in their views of equipment and facilities needed to utilise alternative birth positions. Some midwives complained that there is a shortage of necessary equipment in assisting birthing women, such as a birthing stool, birthing ball and birthing pool in the labour ward. However, other midwives mentioned that currently in the ward there were electronic beds, which also allow the midwives to position the mother in other positions. In contrast, the midwives did not know how to utilise the bed for alternative birth positions:

‘We do not have facilities to use alternative birth positions. I only saw equipment for lithotomy position the lithotomy poles on the beds. I think the main problem is facilities and to buy them can be expensive, and that’s a problem …’ (Participant 4, female, 24 year’s, Professional nurse & 1-year midwifery experience)

‘The facilities are not available and the planning of the unit does not anywhere involve the midwives. The hospital needs to buy convenient birthing chairs, but there is no space in the unit as its already built this way [*and*] doesn’t accommodate birth stools …’ (Participant 7, female, 55 year’s, Advanced Midwife & 8 year’s midwifery experience)

Another participant differed in opinion regarding the availability of facilities:

‘I think the alternative positions can be done, but it depends on the delivery beds sometimes we do not get time to transfer the mother as labour is fast to the delivery beds, and they sometimes deliver on the admission bed. Therefore it becomes difficult [*to*] use alternative positions as the admission beds are not electronic …’ (Participant 5, female, 32 year’s, Professional nurse & 5 years midwifery experience)

#### Communication difficulties between the midwives and the women

The midwives identified the leading cause of communication difficulty as the language barrier that exists among them and the labouring women. The midwives experience difficulty in instructing women to adopt various positions due to the language barrier:

‘It is difficult for us to offer alternative birth positions because most of the times the patients we assist do not know how to communicate in our South African languages or even in English. So it is difficult for women to follow instructions during labour and that hinders us from allowing them to take preferred birth position. If we did not have the problem of [*the*] language barrier, then it would be easier for us to communicate with them …’ (Participant 10, female, 26 year’s, Professional nurse & 4 year’s midwifery experience)

‘We get a lot of women coming from Africa who do not understand English to deliver in our unit. You can’t instruct someone who doesn’t understand you. So with lithotomy position it’s safe because, once they look up maybe you the midwife can look at woman during birth and use sign language to instruct her. With other birth positions like squatting they might be looking down and not hearing what you are saying. So it becomes difficult delivering the woman because of [*the*] language barrier …’ (Participant 11, female, 31 year’s, Professional nurse & 6 year’s midwifery experience)

## Discussion

### Midwives’ perception of alternative birth positions

The colonisation of midwifery training on alternative birth position differed for each midwife. Most of the midwives did not grasp the skill of utilisation of alternative birth positions during undergraduate studies. Thus, the midwives’ preference for lithotomy is underpinned by the lack of correlation of theory and practice of evidence-based positions.

Most midwives prefer to use the lithotomy position because they believe it is easy to manage and is what they are confident using. Limited research has been conducted on reasons midwives prefer the lithotomy position. Literature indicates that midwives should allow women to decide on their birth position of choice irrespective of their own perceived preferences. A study highlighted that women’s involvement in decision-making regarding their labouring process is crucial; it has a profound effect on their birth experiences and satisfaction of the care provided.^13^ The better birth initiative is one of the guidelines used in midwifery practice to guide the best evidence-based labour practices.^20^ It also challenges health care providers to critical thinking and question the standard practices. The better birth initiative encourages health care providers to change the known practices. The initiative also highlights the theme of avoiding harm and respect to women who are being cared for. The midwives should not position women in the lithotomy/supine position because when a woman is placed in this position, there may be reduced blood flow to the uterus, and this interferes with the progress of the second stage of labour. The practice is also degrading and painful; therefore, no matter what the personal preference of the midwives is, the lithotomy birth position needs to be avoided.^20^

Quality maternal and new-born care as highlighted illustrates the three practice categories for all childbearing women and infants within a framework. Firstly, education, information and health promotion should be rendered by midwives. In relation to the study, the midwives need to provide education to birthing women on different alternative birth positions available to them. Secondly, the midwives need to assess, screen and plan the care to be rendered. Lastly, the midwives need to promote normal processes of labour to prevent complications. These categories are most effective when integrated into the health system in the context of effective teamwork, referral mechanisms and sufficient resources.^21^ For midwifery practice to be functionally embedded within the healthcare system, it needs to be available, accessible, acceptable (organisation of care), respectful, understanding and woman-centred (values) and focus on the promotion of physiological processes, strengthening resources and taking a non-interventional stance (philosophy). Midwives and other healthcare providers need to be interpersonally and culturally competent and maintain clarity of roles and responsibilities in their inter-professional relationships.^21^

A model that describes the importance of involving women in their birthing experience is called a women-centred model. The women-centred childbirth model was developed by Maputle.^22^ This model provides an overview description on the women’s experiences of childbirth, and that of attending midwives for managing women during childbirth. The women-centred care model integrates the Batho Pele principles within this model. One of the principles integrated is the Consultation principle, which helps foster more participative decision-making and co-operative relationships between providers and users of public services. The shared decision-making and more active involvement of consumers in their health care could increase consumers’ perceptions of control, which in turn could improve health outcomes.^22^

One of the strategies to enhance mutual participation concerns autonomy; mothers displayed limited information, understanding and awareness of what should be attained during childbirth. This contributed to their inability to make informed choices during childbirth. When limited opportunities were created, mothers become powerless, as evidenced by limited participation, responsibility-sharing, decision-making ability and dependency. When there is an exchange of information and knowledge between the mother and a midwife about childbirth issues and available childbirth options, mothers will become empowered.^22^

The women-centred model promotes women’s informed consent and choice.^23^ The model highlights measures for monitoring different aspects of care quality. It primarily addresses disrespect and abuse; their approach also highlights the interrelationships between the personal experiences of care and the health system. This supports a contextual approach to the quality of care with the focus starting with the woman’s perception and experience of care. This is seen as a central rather than a peripheral component of the quality of care. It was found in this study that midwives, hospital managers and policy-makers diminished women’s choices, due to having a common view that women in labour are unable to make the right decisions and need to be told what to do.^23^

Women value support as suggested by Nieuwenhuijze when highlighting the importance of women valuing the support that maternity care providers can offer. However, they also want to have an influence on the decisions regarding birthing positions in conjunction with maternity care providers. The World Health Organization recommends that women’s birth position choices should be supported during the first and second stages of labour.^3^ A limited number of midwives in the study encouraged the notion of giving women a choice of birth position. Therefore, a clinical decision should be based on available evidence-based literature to enhance women’s positive birthing experiences.

### Barriers to the utilisation of alternative birth positions

Barriers to the utilisation of alternative birth positions are lack of necessary skills and training, lack of facilities and equipment, and communication difficulties between midwives and women. Most of the midwives expressed that they have no skill to conduct birth in an alternative birth position. Literature suggests that skilled birth attendants including midwives need to be placed in labour units to ensure the survival and safety of pregnant women and infants. The allocation of skilled attendants (midwives) in the labour ward will assist in the reduction of maternal and new born mortality rates.^11,24^ In support, two significant strategies that recommend the reduction of maternal mortality: skilled staff in childbirth health facilities and timely access to emergency maternal obstetric care in cases of complications. It has been estimated that Africa needs about one million doctors, nurses and midwives to provide the necessary services needed to achieve the Millennium Developmental Goals, now called Sustainable Developmental Goals.^25^

Consequently, unqualified personnel often provide care, and this affects the quality of services rendered. Therefore, research stipulates the necessity of having skilful birth attendants in labour wards to ensure the achievement of Sustainable Developmental Goal number 3: dealing with ensuring healthy lives and promoting the well-being for all at all ages. The target is to reduce the global maternal mortality ratio to less than 70 per 100 000 live births by 2030. It is important to note that this can be achieved by increasing the number of skilled birth attendants.^12^

In contrast, the midwives varied in their perception of equipment needed in the labour ward. Some midwives verbalised that the environment is already equipped with the necessary apparatus to utilise alternative birth positions such as the beds. A study conducted alludes to that the birth environment (or space) and a woman’s hormone response to her labour affect childbearing.^26^ It has been identified that the ‘bed’ remains a dominant and central feature of most Australian birth rooms, as applicable in the hospital under study. The shortage of necessary equipment leads to the following implications such as staff members being overworked, risk of infections for the midwives, inadequate monitoring and delays in treatment, thus resulting in unnecessary obstetrical complications to women.^24,27^ Therefore, the labour ward under study possesses the necessary equipment to utilise alternative birth positions.

Another highlighted barrier that coexists is the language barrier. The language barrier was emphasised as a communication difficulty resulting from the parties speaking different languages. Studies indicate that the existence of the language barrier has shown to be a threat to the quality of hospital care.^28,29^ The non-English speaking population utilises a growing volume of healthcare services and thus comes into contact with nurses daily. Literature alluded to that some of the adverse events that occur within the maternity units, may be related to the language barrier as a result of communication difficulty between midwife and patient, adverse events reported were associated with language barriers. For example, it was found that the ward personnel end up causing medication errors in those patients who had a language barrier compared to those who did not.^30^

### Limitation of the study

The source of the data was mostly dependent on the midwives of the specific hospital in Tshwane. As such, the findings cannot be transferable as the study is not representative of the entire population.

### Recommendations

This study aimed at exploring factors affecting midwives’ utilisation of alternative birth positions. It is evident that midwives lack skills related to the utilisation of alternative birth positions excluding the lithotomy birth position. The following recommendations emerged:

- It is recommended that midwifery practice be intensified through provision and implementation of evidence-based alternative birth positions. Midwives currently training need to be trained by an advanced skilled expert midwife. Alternative birth positions are the cornerstone to the implementation of evidence-based birth positions that enhance optimal maternal and neonatal outcomes.
- Nursing education institutions should revise the midwifery programme. The programme curriculum should capacitate midwives to teach midwives on the available alternative birth positions and incorporate theory into practice on provision of alternative birth positions.
- Midwives will be capacitated to promote midwifery care that renders women-centred care, which ensures that a woman’s choice and decision during childbirth are enhanced.
- Midwives should provide health education to the birthing women on all birth positions, by using posters and leaflets.
- Hospital management, along with the unit manager and the midwives, should form a team. Team collaboration to formulate a ward protocol on alternative birth positions, based on the evidence-based literature in the guidelines for maternity care in South Africa.
- Lastly, midwives should learn other South African languages and make use of a family companion or interpreter for translation in situations of language barriers.

## Conclusion

Evidence is provided in this study that midwifery practice in the hospital still follows a workplace culture that routinely positions all women in the lithotomy position during labour. Irrespective of the knowledge midwives have on the negative maternal and neonatal outcomes associated with the lithotomy position, they continued utilising this position for their own convenience and overlooked other birthing positions and the women’s preferences. Therefore, the study strongly recommends that midwifery programmes should be designed in such a way that they equip midwives with the necessary skills to utilise alternative birth positions. Furthermore, midwives are encouraged to keep abreast with developments on the provision of alternative birth positions.
